# Antiviral Effects of Green Tea EGCG and Its Potential Application against COVID-19

**DOI:** 10.3390/molecules26133962

**Published:** 2021-06-29

**Authors:** Ying-Qi Wang, Qing-Sheng Li, Xin-Qiang Zheng, Jian-Liang Lu, Yue-Rong Liang

**Affiliations:** 1 Tea Research Institute, Zhejiang University, Hangzhou 310058, China; yqwan@zju.edu.cn (Y.-Q.W.); xqzheng@zju.edu.cn (X.-Q.Z.); 2 Institute of Sericulture and Tea, Zhejiang Academy of Agricultural Sciences, Hangzhou 310000, China; liqs@zaas.ac.cn

**Keywords:** EGCG, tea polyphenol, antiviral, COVID-19, SARS-CoV

## Abstract

(–)-Epigallocatechin-3-*O*-gallate (EGCG), the most abundant component of catechins in tea (*Camellia sinensis* (L.) O. Kuntze), plays a role against viruses through inhibiting virus invasiveness, restraining gene expression and replication. In this paper, the antiviral effects of EGCG on various viruses, including DNA virus, RNA virus, coronavirus, enterovirus and arbovirus, were reviewed. Meanwhile, the antiviral effects of the EGCG *epi*-isomer counterpart (+)-gallocatechin-3-*O*-gallate (GCG) were also discussed.

Tea is a widely consumed non-alcoholic beverage prepared using leaves of tea plants (*Camellia sinensis* (L.) O. Kuntze) [1]. Tea consumption has been proved to benefit human health, among which polyphenol compound tea catechins play important roles, including antioxidant [2], antidiabetes [3], anti-β-Amyloid [4], anti-cancers [5,6], anti-inflammation [7], anti-allergy [8] and antiviral effects [9], among which (–)-Epigallocatechin-3-*O*-gallate (EGCG) is the most abundant component, accounting for more than 40% of the total catechins in green tea [10].

Acute and chronic infectious diseases induced by various viruses has attracted great attention of the world because they cause a serious impact on human health. In the twenty-first century, humans have witnessed three deadly pandemics so far that are associated with novel coronaviruses: SARS, Middle East respiratory syndrome (MERS) and the coronavirus disease 2019 (COVID-19) [11]. The COVID-19 pandemic is an ongoing global pandemic caused by severe acute respiratory syndrome coronavirus 2 (SARS-CoV-2) [12], the World Health Organization (WHO) declared the outbreak a public health emergency of international concern on 20 January 2020. Globally, as of 6:19 pm CEST, 11 June 2021, there have been 174,502,686 confirmed cases of COVID-19, including 3,770,361 deaths [13], making it one of the deadliest pandemics in history. The pandemic has resulted in significant global social and economic disruption, including the largest global recession since the Great Depression [14]. Owing to the short supply of vaccines and specific drugs, finding alternative drugs from natural herbs to circumvent COVID-19 is considered to be a promising strategy for preventing the pandemic [15].

Polyphenols are considered to be a promising antiviral agent against multiple viruses, such as influenza A (H1N1) [16], Ebola virus (EBOV) [17], SARS-CoV (severe acute respiratory syndrome) [18], even SARS-CoV-2 (COVID-19) [15]. EGCG is an abundant polyphenol compound in tea. The in vitro and in vivo inhibitory effects of EGCG on various viruses were summarized in the present review, which will provide a reference for developing antiviral agents from the popular beverage leaves. 

## 1. EGCG against DNA Virus

### 1.1. Effects of EGCG on Hepatitis B Virus

Viral hepatitis is an infectious disease mainly caused by five hepatitis viruses, including hepatitis A, B, C, D and E. According to the “Global health sector strategy on viral hepatitis 2016–2021” formulated by the WHO, viral hepatitis is one of the seven leading causes of death in the world. There are 1.4 million deaths caused by acute hepatitis virus infection and hepatitis-related cirrhosis or liver cancer, of which 47% and 48% of death are caused by hepatitis B virus (HBV) and hepatitis C virus (HCV), respectively. These two hepatitis viruses are highly infectious with great concern around the world.

HBV is a double-stranded DNA virus with a small genome (~3.2 kb) that affects 350–400 million individuals worldwide. HBV is extremely contagious and cannot be completely healed, although interferon (IFN) and nucleotide have played key roles in anti-HBV therapy, which cannot be administered continuously due to side effects and the development of resistance. EGCG has been able to suppress HBV replication and expression through inhibiting HBV promoter transcription, DNA replication, viral incomplete autophagy induction and chronic pathogenesis inhibition [17,18,19,20,21]. 

As a member of the Hepadnaviridae family, HBV’s mRNA synthesis is influenced by liver-enriched transcription factors (LETFs) such as farnesoid X receptor (FXR α), several hepatocyte nuclear factors (HNFs) and retinoid X receptor α (RXR α). By forming heterodimers with RXR α, FXR α plays a key role in lipid biosynthesis and metabolism. A test using fluorescence quenching and affinity binding showed that EGCG was an important transcriptional regulator of HBV genome through interacting with FXR α. When expression plasmids of FXR α and retinoid X receptor α (RXR α) were co-transfected into human embryonic kidney 293 cell line (HEK293), 10–50 μM EGCG could effectively inhibit the transcription of the HBV promoter dose dependently. After 9 days of treatment with 100 μM EGCG, the level of HBV mRNA was decreased to about 20% of control, and the inhibition rates of both HBsAg and HBeAg were more than 99%. These confirmed that the downregulation of the HBV antigen and the decrease in the transcriptional activation of the HBV EnhII/core promoter by FXR /RXR α are mainly due to the interaction between EGCG and FXR α. Therefore, EGCG, an antagonist of FXR α in liver cells, has the potential to be employed as an effective anti-HBV agent. [19]. 

Macroautophagy (hereafter autophagy) is a highly conserved cellular process. Autophagosomes are organelles necessary for HBV replication, while HBV could induce autophagosomes formation. With this mutual promotion procession, cytoplasmic substances are separated into autophagosomes and finally degraded by fusion with lysosomes; thus, autophagy plays an important function in HBV replication. Based on these reasons, controlling the occurrence of viral autophagy is a potential treatment strategy for viral diseases. Zhong et al. [20] found that EGCG induced, but not inhibited, autophagosome formation in hepatoma cells. Different from HBV-induced incomplete autophagic, EGCG could induce a complete autophagic process and opposed incomplete autophagy induced by HBV through impairing lysosomal acidification, which was unfavorable for HBV replication [21]. 

HBV possesses a 3.2 kb relaxed circular partially double-stranded DNA (RC-DNA), which will release into the cytoplasm and converted into covalently closed circular DNA (cccDNA) in the host cell nucleus after infection. Hepatitis B virus e antigen (HBeAg) may promote HBV chronicity by functioning as an immunoregulatory protein [22], which can only be translated from HBV precore mRNA that is solely produced from replicated HBV DNA. Therefore, the precore mRNA and HBeAg level may represent HBV DNA synthesis ability. In an inducible HBV-replicating cell line human hepatocellular carcinomas (HepG2.117), EGCG suppressed the expression of precore mRNA and HBeAg, impaired HBV replicative intermediates of DNA synthesis and inhibited the transformation of RC-DNA into cccDNA through DNA replication mechanism, thereby alleviating the development trend of chronic disease of HBV [23]. 

### 1.2. Effects of EGCG on Herpes Simplex Virus 

Herpes simplex virus (HSV), with global infection rates between 65–90%, is the main cause of genital ulcers, gingivostomatitis and cephalomeningitis, and one can be infected through the mucosa and damaged skin. Previous researches indicated that HSV envelope glycoproteins gB and gD play a key role in mediating viral-cell attachment and fusion. 

Electron microscope study showed that EGCG treatment reduced the activity of gB and gD, while capsid protein labeling was unchanged. Meanwhile, the increasing macromolecular complexes of EGCG with purified HSV-1 envelope glycoproteins gB or gD during incubation proved the viral transmission inhibiting ability of EGCG [24]. 

MST-312, a synthetic telomerase inhibitor of HSV-1, could directly inactivate HSV-1 through interfering with the life cycle of virions at 37 °C [25]. The structure of MST-312 shares moieties related to EGCG. In African green monkey kidney cells (Vero cells), EGCG (0.5–1.0 μM) showed a stronger ability in reducing plaque formation than MST-312 (40–100 μM). Based on the similar structure moieties between MST-312 and EGCG, it seems that the mechanism of EGCG in inhibiting HSV-1 is through suppressing telomerase expression, hence accelerating HSV-1 apoptosis [26]. Thus, EGCG is stable in the pH range found in the vagina and appears to be a promising candidate for use in a microbicide to reduce HSV transmission. 

In vitro testing indicated that theaflavins (TFs), a group of oxidation products of tea catechins in black tea, inhibited viral replication of HSV-1, in which treatment of 50 μM theaflavin-3,3′-digallate (TF-3) for 1 h had an inhibition rate up to 99%. Meanwhile, results of the cytotoxicity assay tested by MTS [3-(4,5-dimethylthiazol-2-yl)-5-(3-carboxymethoxy-phenyl)-2-(4-sulfophenyl)-2*H*-tetrazolium] indicated that theaflavin (TF-1), theaflavin-3-gallate (TF-2) and TF-3 are not toxic to Vero cells at a concentration up to 75 μM. TF-1 andTF-2 showed viral replication inhibition ability during the mitotic phase. Furthermore, TF-2 could suppress inflammation through inhibiting the mRNA expression level of cyclooxygenase-2 (COX-2), tumor necrosis factor A (TNF-A), cellular adhesion molecule 1 (CAM-1) and nuclear factor kappa-B (NF-κB) in vivo [27].

### 1.3. Effects of EGCG on Epstein–Barr Virus

Epstein–Barr virus (EBV) is a human DNA herpesvirus, which is closely related to nasopharyngeal carcinoma, Burkitt’s lymphoma and Hodgkin’s disease [28]. Similar to other hepatitis viruses, EBV has long-term latency or lytic infection in host cells once malignant tumor pathogenesis [29]. 

Immunofluorescence analysis revealed that EGCG (>50 μM) inhibited the expression of EBV lytic proteins, including replication and transcription activator (Rta), zipper transcription activator (Zta) and early antigen-D (EA-D), but had no effect on Epstein–Barr virus nuclear antigen (EBNA-1) expression. Moreover, DNA microarray and transient transfection analyses demonstrated that at concentrations exceeding 50 μM, EGCG blocks EBV lytic cycle by inhibiting the transcription of immediate-early genes in P3HR1 cells, thus inhibiting the initiation of EBV lytic cascade [30]. By decreasing the phosphorylation and activation of extracellular signal-regulated kinase 1/2 (ERK1/2) and Akt, EGCG has been found to inhibit the constitutive lytic infection of EBV at the DNA, gene transcription and protein levels [31]. 

## 2. EGCG against RNA Virus

### 2.1. Effect of EGCG on Human Immunodeficiency Virus 

The acquired immunodeficiency syndrome (AIDS) caused by human immunodeficiency virus (HIV) is an infectious disease transmitted through sexual contact, blood transmission and mother-to-child transmission. HIV uses CD4 lymphocytes as the main target to invade, thoroughly destroying human body immune function, in which partial patients in later stages lost their lives due to other complications resulting from HIV deterioration. Highly active antiretroviral therapy (HAART) is currently the only effective treatment for AIDS. With the help of HAART treatment, the average life expectancy of patients can be extended for decades. Although AIDS cannot be completely healed at this moment, it can be controlled for a long period of time. However, patients treated with HAART exhibited side effects such as drug-induced liver injury and blood toxicity, and some patients died due to complications caused by drug toxicity. Therefore, looking for anti-AIDS drugs with weaker cytotoxicity, fewer side effects and therapeutic effectiveness is still an area worth exploring.

There are studies showing that EGCG has a significant inhibitory effect on transcription and recognition of HIV, which shows the potential for AIDS therapy. Transcription is a crucial step for HIV-1 gene expression in infected host cells. The HIV-1 Tat activates the nuclear factor-kappa B (NF-kappa B) signaling transduction pathway, downregulates intracellular glutathione expression and enhances ROS accumulation, resulting in the promotion of HIV-1 gene expression. Test on HIV-1 Tat protein-activated MAGI cell lines with EGCG showed that EGCG stimulated the expression of Nrf2 and AMPK and inhibited the activation of NF-kB and the accumulation of ROS. The Nrf2 signaling pathway is considered to be the primary target for the prevention of Tat-induced HIV-1 transactivation by EGCG. Additionally, EGCG reduced NF-kappa B activation by inhibiting AKT signaling pathway and activating AMPK signaling pathway. All these lead to the suppression of HIV-1 gene transcription [32].

EGCG could attenuate the infection of HIV to the host cells. When HIV-1 infects the host cells, its outer membrane glycoprotein 120 (gp120) initially binds to the primary receptor CD4 on the host cell surface. Studies using molecular docking, molecular dynamics simulations and binding free-energy analysis revealed that the calculated binding affinity between EGCG and CD4 (EGCG-CD4) was much stronger than that between gp120 and CD4 (gp120-CD4). The favorable binding of EGCG with CD4 can effectively block gp120-CD4 binding [33], blocking the HIV-1 infection to the host cells. This was also verified by magnetic resonance spectroscopy [34]. Semen-derived enhancer of virus infection (SEVI), amyloid fibrils in semen that are formed by proteolytic fragments of prostatic acid phosphatase (PAP248-286 and PAP85-120) and semenogelins (SEM1 and SEM2), could potently enhance HIV infectivity. EGCG could remodel SEVI and PAP248-286 [35] and prevent amyloid fibers formation [36], resulting in attenuation of SEVI enhancement, which exerted a direct antiviral effect on HIV-1 [37].

In addition, EGCG has been proven to suppress HIV strains LAI/IIIB and Bal replication in human peripheral blood mononuclear cells by inhibiting the reverse transcription process of HIV and reducing the mRNA production during viral transcription [38]. Tea polyphenols including EGCG could prevent HIV-1 entry into target cells through blocking membrane fusion mediated by HIV-1 envelope glycoprotein [39]. Because this inhibition can be achieved at physiologic concentrations [39], the natural anti-HIV agent EGCG is a potential candidate as an alternative in HIV-1 therapy.

### 2.2. Effect of EGCG on Hepatitis C Virus 

Hepatitis C virus (HCV), a positive-strand RNA virus with a characteristic of easy mutation, has chronically infected 159,000,000 individuals worldwide [40]. Over 80% of chronic hepatitis type C is caused by HCV infection, which increased the risk for progressive liver diseases, including fibrosis, cirrhosis and hepatocellular carcinoma [41]. Once the symptoms such as anemia and resistance mutations appeared on the patients, the efficiency of the antiviral drugs might be decreased. 

The life cycle of HCV is closely related to lipid metabolism. EGCG impaired the cellular lipid metabolism, showing antiviral activity [42]. In vitro, EGCG (50 μM) inhibited more than 90% HCV infectivity at the early step, especially the entry step of the viral life cycle. EGCG was found to potently inhibit HCV pseudotyped particles entry into host cells through identifying the envelope glycoproteins of HCV, and this was independent of the genotype of HCV. EGCG potently inhibited cell-culture-derived HCV entry into hepatoma cell lines and primary human hepatocytes and blocked cell-to-cell infection by extracellular virions, regardless of the genotype of HCV [43].

EGCG was found to modulate the conformation of heat shock protein 90 (HSP90) [44], a protease that plays a key role in regulating HCV RNA replication. Meanwhile, EGCG potently inhibited the expression of HCV non-structural protein 3 (NS3) and 5B (NS5B); the former plays a decisive role in the transcription and hydrolysis of polyproteins, while the latter modulated RNA polymerization of viruses [45]. EGCG inhibited these proteases by suppressing the hepatitis type C expansion and deterioration through multiple pathways.

In terms of drug synergy, EGCG can significantly improve the efficacy of HCV-specific drugs Sofobuvir and Daclatisvir. Compared to standard care, the incorporated EGCG enhanced drug efficacy and prevented relapse by interfering with the viral entry mechanisms. Meanwhile, the anti-hemolytic and anti-fibrotic activities of EGCG may improve the safety and tolerability of the therapy [46]. 

### 2.3. Effect of EGCG on Influenza A Virus 

Influenza A virus (IAV) is negative-sense RNA segments with eight single-strands, which allows gene re-assortment, while different IAV subtypes coinfect with one single host cell. Gene re-assortment between avian, human and swine influenza viruses caused pandemic influenza repeatedly, among which the acute respiratory disease caused by avian influenza subtype is characterized by high morbidity, onset rapidly and all ages are susceptible.

In vitro, catechin-enriched green tea extract was found to have significant bacteriostatic effects on influenza virus A3 (H3N2) [47]. Docking simulations revealed that green tea catechins with galloyl group fit well into the active pocket of the endonuclease domain and, hence, inhibit influenza A RNA polymerase expression [48]. In vivo testing on H1N1 infected BALB/c mice showed that oral administration of EGCG (40 mg/Kg·d) decreased the mean virus number and mitigated viral pneumonia in the lungs, resulting in dramatical improvement of the survival rates of the infected mice, which was equivalent to oral administration of a positive control drug Oseltamivir (40 mg/Kg·d) [49]. Meanwhile, EGCG inhibited virus particles and the cellular membrane hemifusion events happening by reducing the viral membrane integrity, thereby weakening the cell penetration capacity of the influenza virus [50]. EGCG also inhibited hemagglutination and RNA synthesis in H1N1-infected MDCK cell culture [50]. It strongly inhibited the adsorption of viruses on red blood cells and restricted the growth of avian influenza virus in vivo with a minimum inhibition concentration of 5–10 µM [51]. The antiviral activity is considered to be mediated by its interaction with hemagglutinin (HA)/viral membrane, rendering HA less membrane fusions at the initial stage of infection [51]. Many catechins compounds showed antiviral effects, but EGCG has stronger activity than the others. Tests on H1N2- and H3N2-infected MDCK cells showed that the EC50 (concentration for 50% of maximal effect) of EGCG for inhibiting H1N1 and H3N2 viral replication was 22–28 µM, while the EC50 of (–)-Epigallocatechin (EGC) and (–)-epicatechin-3-*O*-gallate (ECG) was 309–317 and 22–28 µM, respectively [51].

## 3. EGCG against Coronaviruses

### 3.1. SARS-CoV-2 and COVID-19

Coronaviruses are a family of enveloped, single-stranded, positive-strand RNA viruses, widely spreading in many animal species and human beings [52]. Many coronaviruses have adverse effects on humans [53], among which CoV 229E, OC43, NL63 and HKU1 could cause common cold symptoms or severe acute respiratory syndrome. Coronavirus (SARS-CoV), Middle East respiratory syndrome coronavirus (MERS-CoV) and SARS-CoV-2 exerted great harm to humans.

SARS-CoV-2 is an enveloped, positive-sense, single-stranded RNA virus (+ssRNA), with a sequence 29,891 bp. Its genome and subgenome contain at least six open reading frames, encoding four major structural proteins and 12 non-structural proteins, including spike protein (S), membrane protein (M) and 3C-like protease (3-chymotrypsin-like protease, 3CLpro), which are highly related to virus replication and recognition. Its genome sequence shares 79.6% sequence identity to SARS-CoV and is 96% identical at the whole-genome level to a bat coronavirus. It uses the same cell entry receptor–angiotensin converting enzyme II (ACE2) as SARS-CoV [54].

SARS-CoV-2 caused an epidemic of acute respiratory syndrome (COVID-19) [54]. The initial clinical manifestations of COVID-19 include respiratory symptoms such as fever, fatigue and dry cough, accompanied by atypical clinical manifestations such as sore throat, headache and diarrhea [55]. Around one week later, partial patients exhibited difficulty breathing and hypoxia, during which the secretion of intracellular pro-inflammatory factors IL-6, IL-17 and TNF-α increased significantly, and the total number of circulating lymphocytes decreased [55,56], which rapidly deteriorated into acute respiratory distress syndrome (ARDS) [57], sepsis, blood coagulation dysfunction and irreversible metabolic acidosis [58], eventually some severe cases would lead to death.

### 3.2. The Potential Effect of EGCG on SARS-COV-2

**Figure 1:**
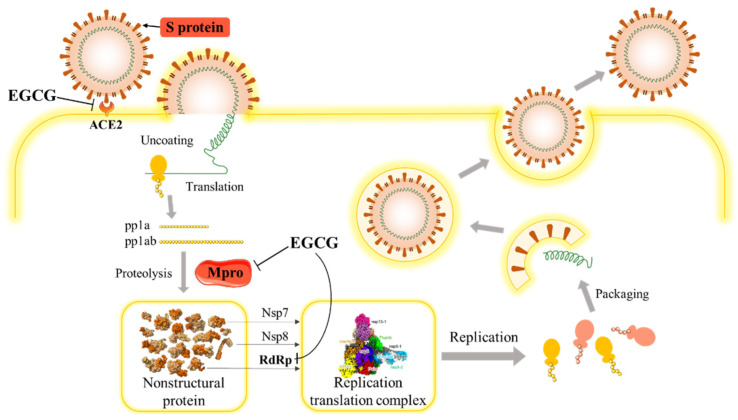
Schematic diagram of SARS-CoV-2 life cycle and the inhibition effects of EGCG.

Cell membrane receptor ACE2 is the binding site for SARS-CoV-2 spike protein, through S protein receptor-binding domain (RBD) on viral membrane identifying with ACE2 and forming RBD–ACE2 complex, by which the SARS-CoV-2 is enabled to get into the host cell where it starts replication [59]. Thus, if a medicine exhibits a strong ability to bind the S protein, or has a strong affinity to ACE2 receptor, which leads to inhibition of RBD–ACE2 complex formation, it would own the potential for repressing viral invading host cells. The inhibition effects of EGCG on SARS-CoV-2 occur through its actions on the ACE2 receptor, the main protease (Mpro, a 3C-like protease) and RdRp (RNA-dependent RNA polymerase) (Figure 1). 

Molecular docking experiments revealed that EGCG had a higher atomic contact energy value, binding energy, Ki value, ligand efficiency and surface area than hydroxychloroquine (HCQ) during binding with the spike protein. There were three binding sites on the spike protein. EGCG can bind with all of the three sites, while HCQ binds only with site III, based on the fact that sites I and sites II are in closer contact with open state location and viral–host contact area. These suggest that EGCG has a stronger ability to inhibit the infection of SARS-CoV-2 to the host cells than HCQ [60]. Molecular docking test on various plant polyphenol compounds showed that EGCG had the highest binding affinity with SARS-COV-2 spike proteins among the 11 tested plant polyphenols (Table 1) [61]. 

Mpro or Nsp5 is an important hydrolase for coronavirus, which is responsible for the proteolysis of coronavirus at the mature stage. The Mpro can hydrolyze the original polyproteins of pp1a and pp1ab from at least 11 conservative cleavage sites to form 12 kinds of new coronavirus non-structural proteins, including RNA polymerase, helicase and methyltransferase. Therefore, Mpro is considered as a prime target for anti-COVID-19 drug development. The test showed that all the eight polyphenol compounds from green tea had a binding affinity toward Mpro, among which EGCG, (–)-epicatechin-3-*O*-gallate (ECG) and (+)-gallocatechin-3-*O*-gallate (GCG) exhibited stronger interaction with catalytic residues His41 and/or Cys145 of Mpro than Mpro inhibitor N3. Molecular dynamics simulations further revealed that these three Mpro–polyphenol complexes were highly stable, experiencing less conformational fluctuations and sharing a similar degree of compactness [62]. In silico studies showed that thearubigins, oxidized compounds of above tea catechins, bound to the Cys145 of Mpro active site and are considered to be a potential pharmacoactive molecule [63]. Molecular docking analysis revealed that multiple bioactive molecules from tea had higher docking scores with SARS-CoV-2 than the proposed repurposed drugs Atazanavir, Darunavir and Lopinavir [64]. In vitro, EGCG and TF, an oxidation product of EGC and (–)-epicatechin (EC), showed inhibitory activity against Mpro of SARS-CoV-2 in a dose-dependent manner; the half inhibitory concentration (IC_50_) was 7.58 μg/mL and 8.55 μg/mL, respectively, without cytotoxicity to HEK 293T cells when the tested concentration being up to 40 μg/mL [65].

The RNA replication and transportation of SARS-CoV-2 can be catalyzed by RNA-dependent RNA polymerase (RdRp), and so, the RdRp is considered to be a potential therapeutic target to SARS-CoV-2 [66]. The molecular dynamic simulation revealed that EGCG, TF-1, TF-2a, TF-2b and TF-3 strongly bound to the active site of RdRp to form highly stable bound conformations with RdRp, resulting in suppression of RdRp expression of SARS-CoV-2 [67]. TFs were also found to bind with RdRp active site strongly in which additional hydrogen bonds were observed between TFs and RdRp. Thus, EGCG and TFs are considered to be potential agents for inhibiting SARS-CoV-2 RdRp [68]. 

Severe COVID-19 patients could have various complications, such as adverse respiratory disorders (ARDS), septic shock, coagulopathy and other life-threatening status. SARS-CoV-2 binds to ACE2 protein of intestinal cell and alveolar cell membrane, causing respiratory and homeostatic disorders, producing a large number of pro-inflammatory factors and eventually developing sepsis. Among them, the rapid progression from acute respiratory failure to sepsis is closely related to the release of high mobility group box-1 protein (HMGB1), a highly conserved protein stored in the nucleus. When immune cells are stimulated by bacterial endotoxin, lipopolysaccharide or cytokine endotoxin, HMGB1 will be released into the extracellular environment to promote inflammatory cytokines synthesis. Intraperitoneal administration of EGCG protected mice against lethal endotoxemia and rescued mice from lethal sepsis even when the first dose was given 24 h after cecal ligation and puncture. EGCG stimulates autophagy and reduces cytoplasmic HMGB1 levels in endotoxin-stimulated macrophages [67]. The protection against lethal sepsis mediated by EGCG was partly impaired by co-administration of an autophagy inhibitor, chloroquine [69]. Furthermore, green tea extract containing EGCG can also reduce the release of HMGB1 induced by endotoxin. When the concentration of green tea extract was as low as 10 μg/mL, HMGB1 could be completely inhibited in macrophages with no evidence of cytotoxicity [69]. However, the kinetic mechanism of EGCG inhibiting the release of HMGB1 has not been clear. The possible reason is that EGCG can bind to lipid raft-related receptors [70], while macrophages rely on the lipopolysaccharide complex to transfer HMGB1 to extracellular environment for pro-inflammatory cytokines synthesis (Figure 1). 

## 4. Effects of EGCG on Enteroviruses

Enteroviruses belong to the *Picornaviridae* family, which can cause respiratory tract infections, skin rashes and severe neurological complications. Most common human enteroviruses are poliovirus (PV), coxsackie B virus (CVB), echovirus and new enterovirus. 

Viral myocarditis refers to a disease in which viruses invade the heart, with myocardial inflammatory lesions as the main manifestation, and is mainly caused by coxsackievirus B3 (CVB3) infection. The clinical manifestations of the disease are differentiated, in which some patients will gradually evolve into dilated cardiomyopathy (DCM), the main cause of sudden death in adolescents. It was proved that although EGCG cannot downregulate the expression of IL-6, TNF-α and other inflammatory cytokines, it can significantly inhibit CVB3 virus replication and downregulate CVB3 and adenovirus receptors expression, thereby alleviating the pathological symptom of cardiomyocytes [71].

EV71 is one of the main pathogens causing hand–foot–mouth disease in infants. The virus is highly contagious and has a high pathogenicity rate. There is a high probability of complications in the central nervous system after infection with EV71 with no specific drug to cure, clinically only supportive therapy can be taken for patients. It was shown that EGCG and its *epi*-isomer GCG had a significant inhibitory effect on the replication of EV71 in Vero cell line, resulting in a marked reduction in the progeny virus infection. The mechanism is considered to be related to its regulation of the oxidative stress of host cells [72]. 

## 5. Effects of EGCG on Arboviruses

*Arboviruses* (arthropod-borne virus), including West Nile virus (*Flaviviridae*), Dengue virus (*Flaviviridae*), Chikungunya virus (*Togaviridae*), etc., are widely spread by arthropod vectors, most commonly, mosquitoes, ticks and sand flies. Chikungunya caused by Chikungunya virus (CHIKV) is characterized as fever, skin rash and joint pain, which has been prevalent in Africa and Southeast Asia for decades. Zika virus (ZIKV) was widely spread in Polynesia in 2013, although only about 20% of patients exhibited mild fever, joint pain and other symptoms, which can self-heal; during ZIKV outbreak, the occurrence of microcephaly in newborn babies increased by 20 times than before [73]. Dengue viruses are mainly spread in tropical and subtropical regions, with typical clinical manifestations of high fever, headache, severe aches and lymphadenectasis. 

Research showed that EGCG not only inhibited CHIVK viral replication but also showed synergistic effects with the drug Suramin against CHIKV [74]. Testing on CHIVK infected U2OS cells showed that EGCG enhances the antiviral activity and anti-inflammatory effects of Suramin through inhibiting the viral RNA replication, resulting in the cytopathic effect of CHIKV [74]. EGCG was shown to prevent virus attachment and entry to the cells [74], inhibiting intercellular infection in the early stage of infection [75] (Table 2). In Vero cells, EGCG (>100 μM) could inhibiting more than 90% ZIKV entry [73]. EGCG showed a directly pH-independent viricidal effect on WNV particles [76]. 

ZIKV NS3 helicase, an enzyme performs an important role in viral replication by unwinding RNA after hydrolyzing NTP. EGCG interacts with NS3 helicase at the ATPase site and RNA binding site, leading to inhibition of ZIKV NS3 helicase activity. EGCG is considered as a powerful molecule against ZIKV and other flaviviruses [79].

## 6. Antiviral Effects of EGCG *epi*-isomer GCG

EGCG is the most abundant polyphenol in fresh tea leaves on cultivated tea plants (*Camellia sinensis*). GCG, an *epi*-isomer counterpart of EGCG, is usually not detected in the fresh tea leaves but in dry tea because EGCG is epimerized into GCG under heating and illumination or extreme pH conditions during tea processing and storage. There were studies that revealed that GCG showed similar antiviral effects as its *epi*-isomer counterpart EGCG. Testing on enterovirus 71 (EV71) showed that GCG, similar to EGCG, significantly reduced the titer of EV71 progeny virus in a dose-dependent manner. Treatment with 10 μM GCG and EGCG resulted in 56% and 54% decreases in titer of progeny virus, respectively. As their concentration was increased to 25 μM, the virus yields were further decreased to approximately 5% of control. The antiviral effect of GCG and EGCG was associated with their cytoprotective effect. Testing showed that the viability of Vero cells infected with EV71 at a moi of 1.25 was 12.0 ± 1.3%. Treatment with 25 μM GCG or EGCG increased the viability of infected cells by approximately five-fold [72]. An in silico docking and molecular dynamics simulation study showed that GCG and EGCG have the interaction with one or both of the catalytic residues His41 and Cys145 from catalytic dyad of Mpro of SARS CoV-2, with binding energy −9.0 and −7.6 kcal/mol, respectively. Estimations of binding free energy using the MM-GBSA method revealed that the Mpro–GCG complex (−53.54 kcal/mol) is relatively more stable than Mpro–EGCG complex (−43.56 kcal/mol) [62]. Green tea EGCG and GCG are considered to be potent anti-COVID-19 drug candidates.

## 7. Conclusions

EGCG, as the major secondary metabolite polyphenol in *Camellia sinensis*, has been confirmed to be a multifunctional bioactive molecule with potential for anti-infective, anti-proliferation and antiviral effects. In this review, the antiviral effects of EGCG on DNA, RNA, coronaviruses and other viruses were summarized and discussed. EGCG acts antiviral functions in different stages of infection for both nuclear viruses and cytoplasmic viruses, and so is considered to be a potential alternative agent for multiple viral diseases (Table 2). 

EGCG is shown to be able to bind strongly with many molecules in viruses, especially protease and protein; therefore, it influences their functional activities. Through attaching with virion surface or the receptors on the host cell membrane, EGCG disturbs the interaction between viral and host cells. EGCG suppresses viral genome replication and viral protein expression, inactivating viral activity and inhibiting pro-inflammatory factor promotion. Besides, EGCG exhibits synergistic effects on several antiviral-specific drugs via reducing toxicity and enhancing the efficacy of drugs, increasing the resistance of cells to drugs. 

**Figure 2:**
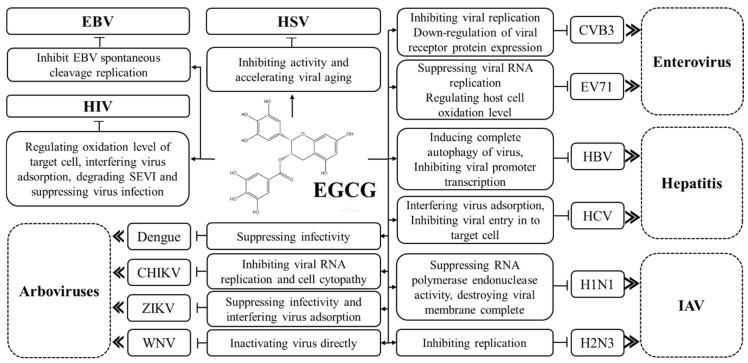
Inhibition effects of EGCG on viruses.

COVID-19 caused by SARS-CoV-2 has become the most threatening disease in the past few decades and caused 3.77 million death globally. Although SARS-CoV-2 vaccination started in January 2021, the efficacy of the vaccine in avoiding repeat infection remains to be confirmed. A series of molecular docking analyses showed that EGCG can prevent SARS-CoV-2 entry into target cell through inhibiting RBD on viral membrane identifying with ACE2, inhibiting viral start replication via suppressing Mpro activity (Figure 1 and Figure 2). EGCG can also play an inhibitory role in complex diseases caused by COVID-19, such as sepsis and coagulation dysfunction. 

The antiviral effects of EGCG on DNA and RNA viruses have been proven in in vitro experiments abundantly, and further studies should be focused on the effects and mechanism of EGCG in vivo and clinical tests. Although the anti-inflammatory effects of EGCG on COVID-19 have been shown by molecular docking experiments, it needs to be confirmed in vivo. It is also interesting to investigate if EGCG has a synergistic effect on COVID-19 vaccines in the future studies.

## Figures and Tables

**Table 1:** Binding energy between polyphenols and SARS-COV-2 spike proteins.

Compound	Total Binding Energy (kcal/mol)	Van der Waal’s Force (kcal/mol)	H-Bond Energy (kcal/mol)	AverCon Pair (kcal/mol)
EGCG	−130.566	−91.7244	−38.8417	25.7273
Curcumin	−115.198	−87.5695	−27.6288	26.963
Apigenin	−108.614	−82.1108	−26.503	33.9
Chrysophanol	−107.385	−90.5916	−16.7935	36.7895
Emodin	−105.462	−87.2314	−18.2303	32.85
Zingerone	−102.184	−77.9523	−24.2321	26.9524
Gingerol	−98.0333	−84.1818	−13.8515	27.6667
Ursolic acid	−89.9499	−72.2658	−17.6841	23.5714
Ajoene	−74.2819	−68.2819	−6	32.4615
Aloe emodin	−69.2503	−69.2503	0	29.2308
Allicin	−62.4326	−62.4326	0	40.3333
Diallyltrisulfide	−53.2872	−53.2872	0	39.2222

**Table 2:** Inhibitory effects of EGCG against viruses.

Virus	Cell Line	Effect	Reference
CHIKV	U2OS	Inhibiting viral RNA replication	[75]
CHIKV	HEK 293T	Blocking viral entry into target cells	[74]
CVB3	Vero	Inhibiting viral RNA replication, improving the survival rate of infected cells	[72]
EBV	B95.8	Down regulating RNA synthesis, inhibiting EBV lytic protein expression	[31]
HBV	HepG2.2.15	Inhibiting HBs Ag and HBe Ag secretion	[21]
HBV	HepG2.2.15	Opposing HBV-induced incomplete autophagy, reducing HBV replication	[20]
HBV	HepG2.N10	Interfering core promoter transcription	[19]
HBV	HepG2.117	Inhibiting RNA, DNA and cccDNA synthesis	[23]
HCV	Huh7.5	Interfering virus adsorption, preventing cell-to-cell transmission	[43]
HCV	Huh7.19	Inhibiting viral entry in to target cell	[42]
HIV	MAGI	Reducing Nf-kB expression and suppressing HIV-1 gene transcription	[32]
HIV	peripheral blood lymphocytes	Inhibiting viral replication	[77]
HIV	HeLa-CD4-LTR-beta-gal	Suppressing viral infection ability, inhibiting viral reverse transcription	[38]
HIV	THP-1	Inhibiting viral transcription	[78]
HSV	Vero, CV-1	In-activating viral	[24]
ZIKA	Vero E6	Inhibiting viral entry in to target cell	[73]
H1N1	HEK 293T	Destroying viral membrane integrity, preventing viral adsorption to cell surface	[50]
H1N1,	MDCK	Inhibiting erythrocyte agglutination and viral replication	[51]

## Data Availability

Not applicable.
